# Laser Irradiation and Property Correlation in Double-Lasing Processes on Laser-Induced Graphene Electrodes

**DOI:** 10.3390/nano15050333

**Published:** 2025-02-21

**Authors:** Tran Quoc Thang, Joohoon Kim

**Affiliations:** 1 Department of Chemistry, Research Institute for Basic Sciences, Kyung Hee University, Seoul 02447, Republic of Korea; tqthang2903@khu.ac.kr; 2 KHU-KIST Department of Converging Science and Technology, Kyung Hee University, Seoul 02447, Republic of Korea

**Keywords:** laser-induced graphene, double-lasing irradiation, enhanced electrochemical property, irradiation and property correlation, electrochemical sensing

## Abstract

The fabrication of laser-induced graphene (LIG) electrodes by direct laser writing techniques has received considerable attention due to its simplicity, versatility, and cost-effectiveness for electrochemical applications in both sensing and energy storage. In general, a single-lasing irradiation process is used to prepare LIG electrodes. However, the intrinsic features of LIG can be further improved by taking advantage of additional lasing processes, even without any chemical treatments. In this work, we investigated the potential enhancement of LIG’s electrochemical performance through a double-lasing irradiation process. This process does not require any chemical modification of the LIG to improve its electrochemical performance. Importantly, we revealed the correlation between laser irradiation and the properties of LIG electrodes prepared through the lasing process. We evaluated the characteristics of LIG electrodes prepared by the single-lasing and double-lasing irradiation regarding their microstructures and electrochemical features, including the sheet resistance (R_S_), specific areal capacitance (C_A_), peak-to-peak separation (Δ*E*_P_), and peak current. The double-lasing LIG exhibited improved electrochemical properties, especially low R_S_ and Δ*E*_P_ values. This improvement results from a higher degree of graphitization, making them advantageous for developing electrochemical sensors. This was demonstrated by the improved electrochemical sensing of H_2_O_2_ using the double-lasing LIG.

## 1. Introduction

Direct laser writing techniques on polymer substrates provide a useful tool for patterning conductive laser-induced graphene (LIG) on non-conductive substrates. The techniques allow the graphene generation and patterning process to occur simultaneously without any masks or chemicals, thus reducing production costs and chemical wastes. The generated LIG inherits traditional graphene synthesized from wet chemical reactions with superior characteristics such as good electrical conductivity (25 S/cm^−1^), high thermal stability, and high porosity (340 m^2^/g) [1]. The LIG has thus emerged as an attractive material used in a wide range of electrochemical applications, including supercapacitors [1,2,3,4,5,6,7], batteries [8,9,10], and electrochemical sensors [11,12,13,14,15,16]. This novel material was first discovered by Tour et al. in 2014. They successfully fabricated LIG-based supercapacitors from polyimide film as a carbon substrate using a CO_2_ laser system with specific areal capacitances of more than 4 mF/cm^2^ [1].

The intrinsic features of LIG can be further enhanced by chemical modification depending on the applications. For instance, heteroatom or metal-based doping in the graphene lattice is a feasible technique for creating electrochemically active sites that adjust the electronic band structure of graphene, resulting in more efficient electrocatalysts for energy storage applications. Tour et al. demonstrated that metal or metal oxides can be incorporated in LIG with a facile lasing process to fabricate oxygen reduction reaction (ORR)-active Co/Mn and oxygen evolution reaction (OER)-active Ni/Fe in rechargeable Zn–air batteries [9], oxygen electrocatalysts Co_3_O_4_/LIG [10], or bifunctional catalyst MnO_2_/LIG as cathode catalyst in quasi-solid-state Li-O_2_ [8]. One common strategy to modify LIG is forming LIG before the drop-casting metal precursors on the first LIG layer; then, the second lasing irradiation is applied to convert metal ions into the respective metal or metal oxide on LIG. Nevertheless, only a few papers have reported multiple lasing steps for bare LIG without any chemical modifications. One way to perform multiple lasing steps on bare LIG is the defocus method [2,17] or by increasing the number of lasing scans [18,19,20,21]. However, few studies have explored the relationship between laser irradiation and the properties of LIG during the multiple-lasing processes. For instance, Tour and coworkers demonstrated the formation of LIG on various substrates including cloth, paper, potato skins, coconut shells, and cork through a multiple-lasing process [2]. Park and coworkers also applied the multiple-lasing process on paper to fabricate LIG electrodes for the production of resistors and capacitors as a cost-effective and environmentally friendly approach [18]. However, they did not provide detailed studies exploring the correlation between the multiple-lasing process and the electrochemical properties of LIG.

Herein, our report discusses the correlation between LIG properties and multiple-lasing processes. Specifically, after optimizing a single-lasing irradiation, we performed a second lasing to improve the electrochemical features, especially sheet resistance (R_S_), specific areal capacitance (C_A_), peak-to-peak separation (Δ*E*_P_), and the peak current of LIG. This investigation aimed to explore the correlation between laser irradiation and the resulting properties in both single- and double-lasing processes. Our results highlight the significance of fully utilizing the lasing process without requiring any chemical modification to enhance the electrochemical characteristics of LIG. This would pave the way for a more environmentally friendly fabrication process and less chemical waste during the lasing process.

## 2. Experimental

### 2.1. Chemicals and Materials

Potassium hexacyanoferrate (III) (K_3_[Fe(CN)_6_]), sodium phosphate dibasic heptahydrate (Na_2_HPO_4_·7H_2_O), sodium phosphate monobasic dihydrate (NaH_2_PO_4_·2H_2_O), and sodium hydroxide (NaOH) were purchased from Sigma–Aldrich, Inc. (St. Louis, MO, USA). Anhydrous sodium sulfate (Na_2_SO_4_), potassium chloride (KCl), hydrochloric acid (HCl), and hydrogen peroxide (H_2_O_2_, 35%) were bought from Daejung Chemicals, Inc. (Gyeonggi, Republic of Korea). Polyimide (PI) film (ST-8357BH) and conductive silver paste (ELCOAT P-100) were obtained from Daehyun ST Co. (Gyeonggi, Republic of Korea) and JIN Chemical Co. (Gyeonggi, Republic of Korea), respectively. Deionized (DI) ultrapure water (18.2 MΩ·cm) was achieved using an ultrapure water system (Aquapuri 5 series, YOUNGIN Chromass Co., Ltd., Gyeonggi, Republic of Korea) and was used to prepare aqueous solutions.

### 2.2. Fabrication of LIG Electrode

**Figure 1:**
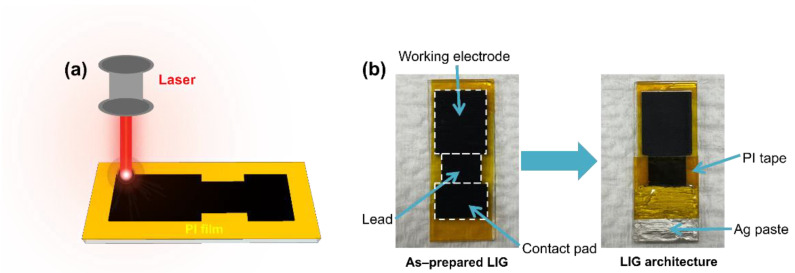
(**a**) Schematic diagram illustrating the formation of an LIG pattern from PI. (**b**) Photographs demonstrating the process of fabricating an LIG electrode architecture.

Laser scribing was performed on PI films (thickness of 50 μm) with a computer-controlled CO_2_ IR laser system (Coryart, C40, Gyeonggi, Republic of Korea, laser wavelength of 10.6 μm) to prepare an LIG pattern designed using AutoCAD. As illustrated in Figure 1, a PI film was adhered to a piece of glass (10 × 25 mm^2^) and subsequently cleaned with acetone, ethanol, and DI water using tissue. The laser scribing was then conducted on the PI by adjusting the laser power between 1 and 100% of 60 W, along with a scanning speed ranging from 10 to 400 mm/s while maintaining a fixed pulse per inch (ppi) of 1000 ppi. The lasing conditions are denoted as D/S–scanning speed–laser power, where D stands for double-lasing irradiation and S for single-lasing irradiation. For instance, S–130–13 indicates single-lasing irradiation on PI at a scanning speed of 130 mm/s and a laser power of 13% of 60 W. To fabricate an LIG electrode architecture from the formed LIG pattern, silver paste was applied to a contact pad area to ensure electrical connectivity. Additional PI tape was applied over the contact pad and lead areas to define the working electrode area (8 × 10 mm^2^) on the LIG pattern.

### 2.3. Characterization

Scanning electron microscopy (SEM) and transmission electron microscopy (TEM) were used to observe the morphologies of LIG using a PicoEye–100 (ModuleSci Co., Daejeon, Republic of Korea) and a JEM-2100F (JEOL, Tokyo, Japan), respectively. Raman spectra were obtained using an inViaTM confocal Raman microscope (Renishaw Ltd., Wotton-under-Edge, UK) with a laser power of 0.5 mW and a wavelength of 532 nm. From the Raman spectra, we calculated the crystalline size along the *a*-axis (*L_a_*) of LIG using the intensity ratio between the G and D peaks (*I_G_*/*I_D_*). The *L_a_* value is determined using Equation (1) [22]:

(1) $$L_{a}=\left(2.4\times 10^{-10}\right)\times \lambda_{l}^{4}\times \left(\frac{I_{G}}{I_{D}}\right)$$

where *λ_l_* is the wavelength of the Raman laser (532 nm). X-ray diffraction (XRD) patterns were recorded using an X’Pert Pro MRD X-ray diffractometer (Malvern PANalytical Ltd., Worcestershire, UK) that utilized CuKα radiation (λ = 0.154 nm). Fourier transform infrared (FT-IR) spectroscopy was conducted to record changes in the chemical features of LIG during the lasing process using an Alpha II (Bruker Corp., Billerica, MA, USA). X-ray photoelectron spectroscopy (XPS) was performed using a K-Alpha X-ray photoelectron spectrometer (Thermo Scientific, Waltham, MA, USA) with Al-Kα source. Sheet resistance (R_S_, in Ω/sq) values were measured using a RC2175 R-CHEK surface resistivity meter (EDTM Inc., Toledo, OH, USA) with a region of interest (ROI) of 4 × 20 mm^2^. All electrochemical measurements were performed using a WaveDriver 200 potentiostat (Pine Instrument Co., Grove City, PA, USA) with a standard three-electrode electrochemical cell comprising an LIG electrode, Ag/AgCl (3 M KCl) reference electrode, and Pt wire counter electrode. A conductive silver paste was applied on the clamp part of the LIG electrode to improve the electrical connection between the electrode and the crocodile clamp. The specific areal capacitance (C_A_, mF/cm^2^) was determined from cyclic voltammograms (CVs) using Equation (2) [1]:

(2) $$C_{A}=\frac{1}{2\times S\times v\times (V_{F}-V_{I})}\int_{V_{I}}^{V_{F}}I\left(V\right)dV$$

where S is the surface area (0.8 cm^2^) of LIG electrodes; v is the scan rate (V/s); V_F_ and V_I_ are the final and initial potential (V); and $\int_{V_{I}}^{V_{F}}I\left(V\right)dV$ is the integrated area from CVs. For the electrochemical sensing of H_2_O_2_, chronoamperometric measurements were conducted using various concentrations of H_2_O_2_ in N_2_-saturated 0.1 M phosphate buffer (pH 7.4).

## 3. Results and Discussion

The formation of LIG generally happens through photothermal or photochemical processes [23]. Photochemical mechanisms depend on the incident laser, providing sufficient energy for overcoming electronic excitation, which leads to the breakage and formation of chemical bonds [23]. On the other hand, photothermal mechanisms are based on the localization of heat from the incident laser energy, which breaks and rearranges bonds [23]. Therefore, in our present study, the formation of LIG was induced by the photothermal effect, resulting from the long wavelength (10.6 μm) of the CO_2_ laser used as opposed to the photochemical process that occurs with short-wavelength laser systems. The energy from the CO_2_ laser can cause localized heating, leading to the breaking of bonds such as C–O, C=O, and N–C in PI substrates [1]. As a result of this photothermal process, gas is released, and the atoms rearrange themselves into a graphitic structure. The gasses released help prevent the oxidation of LIG and contribute to creating a porous microstructure [1,24].

### 3.1. LIG Formed Through a Single-Lasing Process

**Figure 2:**
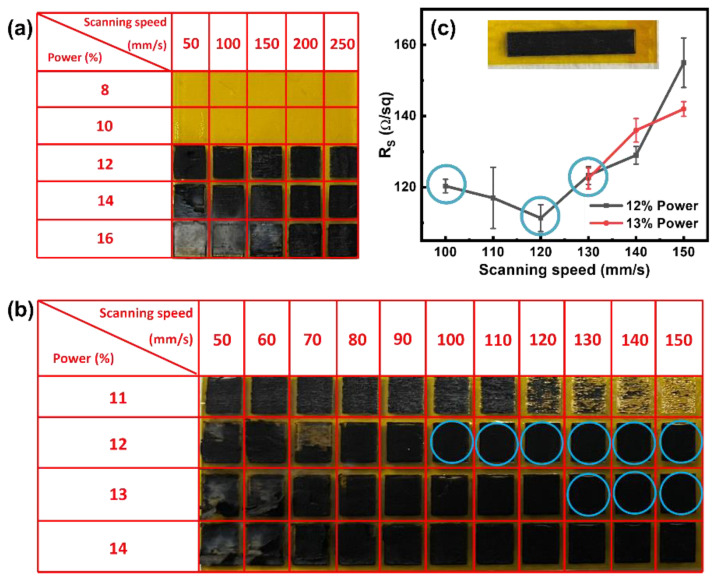
Physical appearance of LIG formed through single-lasing processes under (**a**) wide-range and (**b**) narrow-range lasing conditions. (**c**) Sheet resistance (R_S_) of the LIG as a function of scanning speed and power used. Inset is an optical image of an LIG, showing the dimensions of the LIG used for the R_S_ measurement (20 × 4 mm^2^).

To optimize the single-lasing conditions, including laser power (%) and scanning speed (mm/s), we quickly evaluated the physical appearance of LIG prepared by lasing a 3 × 3 mm^2^ rectangle on PI film. As shown in Figure 2a, we observed that LIG formation did not occur at power levels between 8 and 10%. LIG formation became noticeable at power levels between 12 and 16%, but at 16%, the power was relatively high and caused physical damage, such as peeling off, even at high scanning speeds (e.g., 250 mm/s). Therefore, we identified the power levels of ~12 and ~14% as the lower and upper limits for LIG formation on the PI film, respectively. Next, we narrowed the scanning speed to a range of 50 and 150 mm/s to determine the optimal condition. As illustrated in Figure 2b, we screened the different combinations of laser power and scanning speed conditions while observing the physical changes in the formed LIG during the single-lasing process. We could start the graphitization on the PI film at 11% power, but as the scanning speed increased, we had to use a higher threshold power. These results indicated that it was essential to balance the laser power and scanning speed to obtain well-formed LIG. We evaluated the sheet resistance (R_S_) of nine LIG samples (marked by blue circles in Figure 2b) due to their uniform shapes and absence of peeling off. The results show a volcano-type trend in R_S_ values for the LIG samples obtained at a laser power of 12%, varying the scanning speed from 100 to 150 mm/s, as presented in Figure 2c. The negative slope in the trend of R_S_ obtained from 100 to 120 mm/s suggests that the lasing process reduced R_S_ values as the scanning speed increased, leading to the generation of high-quality LIG films with a high degree of graphitization. However, when the scanning speed was higher than 120 mm/s, the slope was positive. This indicates that thermal power was not provided sufficiently and dissipated too fast due to the high scanning speed. As a result, low-quality LIG with high R_S_ values were produced. With a higher power of 13%, we observed smaller increases in R_S_ values compared to those of 12% at scanning speeds between 130 and 150 mm/s. This suggests that sufficient thermal power was provided to induce LIG formation at a laser power of 13%, but R_S_ values still increased, presumably due to inadequate oxidation that deteriorated LIG layers as the scanning speed increased. Among the nine LIG samples, three LIG samples (i.e., S-100-12, S-120-12, and S-130-13, marked by blue circles in Figure 2c) exhibited the smallest R_S_ and the least deviation in R_S_, indicating appropriate LIG formation. These three samples were thus selected for further investigation of their electrochemical features. It is worth noting that their R_S_ values are comparable with those reported previously with similar CO_2_ laser systems [12].

**Figure 3:**
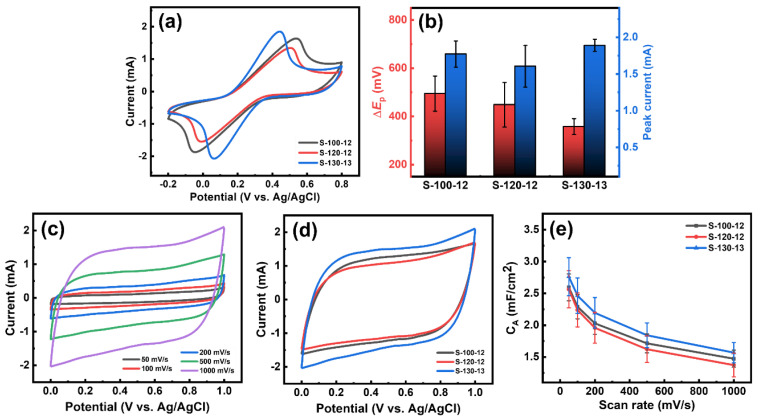
(**a**) CVs of 10 mM K_3_[Fe(CN)_6_] in 1 M KCl for S-100-12, S-120-12, and S-130-13 at a scan rate of 50 mV/s. (**b**) Peak-to-peak separation (Δ*E*_P_) and peak current values obtained from the CVs for each LIG. (**c**) CVs of S-130-13 in 1 M Na_2_SO_4_ at scan rates from 50 to 1000 mV/s. (**d**) CVs of S-100-12, S-120-12, and S-130-13 in 1 M Na_2_SO_4_ at a scan rate of 1000 mV/s. (**e**) Specific areal capacitance (C_A_) calculated from the CVs for each LIG as a function of scan rates.

Figure 3a shows the CVs of [Fe(CN)_6_]^3−^ obtained with S-100-12, S-120-12, and S-130-13 electrodes. The measurements were taken in the potential range of −0.2 to 0.8 V at a scan rate of 50 mV/s. Distinct redox peaks of [Fe(CN)_6_]^3−^ were clearly observed with all three electrode samples. The electrochemical performance of the three electrodes was assessed by measuring the peak-to-peak separation (Δ*E*_P_) and peak current. As shown in Figure 3b, S-130-13 exhibited a slightly improved faradaic peak current of 1.89 ± 0.08 mA compared to S-100-12 and S-120-12. At a fixed scan rate of 50 mV/s, the Δ*E*_P_ values were 494.6 mV for S-100-12, 448.5 mV for S-120-12, and 357.8 mV for S-130-13. These values indicate the quasi-reversible electrochemical behavior of [Fe(CN)_6_]^3−^ on the three electrodes, but the smallest Δ*E*_P_ value for S-130-13 suggests that it is a promising candidate for use in electrochemical sensors and energy storage devices.

In addition, the electrochemical capacitance of S-100-12, S-120-12, and S-130-13 was evaluated using cyclic voltammetry at various scan rates ranging from 50 to 1000 mV/s in a 1 M Na_2_SO_4_ solution. All electrode samples exhibited symmetric quasi-rectangular shapes, indicating typical electrical double-capacitive-type charge storage behavior (Figure 3c,d and Figure S1a,b) [25]. This behavior results from the accumulation of electrostatic charges at the electrode/electrolyte interface through the adsorption of electrolyte ions [26]. Their symmetric quasi-rectangular shapes remained stable even at high scan rates of 1000 mV/s, demonstrating the high stability of the electrodes, which is desirable for energy storage applications. Figure 3e shows the specific areal capacitance (C_A_) values of the three electrodes, evaluated at the scan rates ranging from 50 to 1000 mV/s. The C_A_ values decreased as the scan rates increased, which is attributed to the limitations in the charge mobility of the electrolyte. Specifically, at lower scan rates, the electrolyte ions have sufficient time to penetrate the inner pores of the LIG electrodes, resulting in greater charge accumulation at the electrode/electrolyte interface and enhanced capacitive behavior. In contrast, higher scan rates lead to insufficient charge mobility of the electrolyte at the porous surface of the LIG electrodes. This resulted in reduced charge accumulation, causing the C_A_ values to decline as scan rates increased [27,28]. In particular, the C_A_ value of S-130-13 was measured to be 2.76 ± 0.30 mF/cm^2^ at a scan rate of 50 mV/s. This value is comparable to or exceeds those of chemically modified LIG, as shown in Table S1 (Supporting Information). At a higher scan rate of 1000 mV/s, the capacitance still retained a value of 1.57 ± 0.16 mF/cm^2^, which is 56.9% of the value obtained at 50 mV/s.

**Figure 4:**
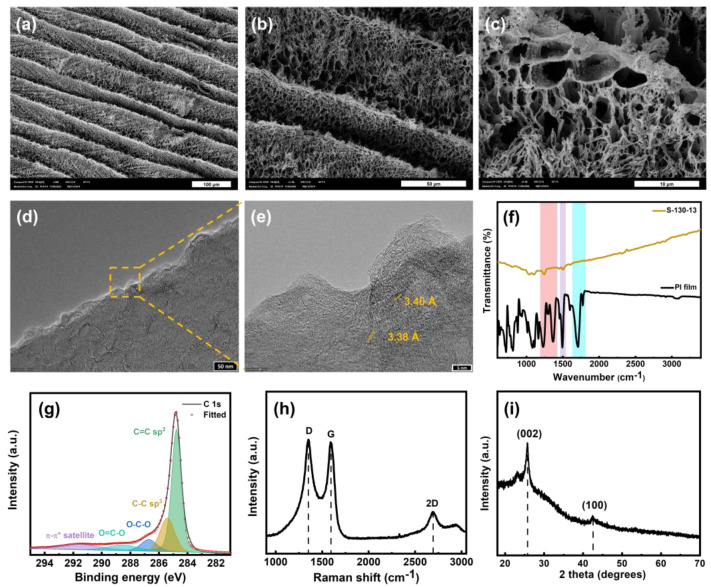
SEM images of S-130-13 at different magnifications of (**a**) 300×, (**b**) 1000×, and (**c**) 5000×. (**d**,**e**) TEM images of S-130-13 at different magnifications. The scale bar in panel (**d**) measures 50 nm, while the scale in panel (**e**) measures 5 nm. (**f**) FT-IR spectra of PI film and S-130-13. (**g**) Deconvoluted C 1s XPS spectrum of S-130-13. (**h**) Raman spectrum of S-130-13. (**i**) XRD pattern of S-130-13.

Figure 4a–c displays the SEM images of S-130-13. Additional SEM images of other single-lasing LIG samples are presented in Figure S2a–d (Supporting Information). These images reveal a foam-like morphology of the LIG with diverse pore sizes. This porous structure is the result of rapid gas emissions during the lasing process. As shown in Figure 4c, the LIG foam consists of interconnected networks of graphene fibers. These pores allow electroactive species to infiltrate the three-dimensional network, enhancing the electrochemical response. As shown in Figure 4d,e, the TEM images of S-130-13 also reveal a rippled surface morphology on the graphene sheets, which was formed during thermal expansion caused by the laser irradiation. The lattice spacing between successive layers of graphene sheets was estimated to be ~3.4 Å, which is consistent with the previous literature [1]. Figure 4f demonstrates significant changes in the chemical structures between the original PI film and S-130-13. The FT-IR spectrum of the PI film exhibits characteristic peaks at 1776, 1710, 1494, and 1372/1226 cm^−1^, which correspond to C=O symmetric stretching, C=O asymmetric stretching, C=C stretching, and C–N stretching, respectively [29,30,31]. After the laser irradiation process, we observed a broad shoulder overlapping with small peaks between 1000 and 1700 cm^−1^, indicating significant changes in chemical structures. Specifically, the laser irradiation diminished most of the characteristic peaks, suggesting a substantial reduction in oxygen-containing functional groups compared to the original PI film. This finding is consistent with previous reports [32,33,34]. The XPS survey spectrum of S-130-13, shown in Figure S3a (Supporting Information), displays a dominant C 1s peak located at ~284.8 eV. In contrast, the O 1s peak located at ~533 eV is significantly smaller in comparison to the C 1s peak. This observation confirms the substantial reduction in oxygen-containing groups after the laser irradiation process [1]. Furthermore, Figure 4g shows a deconvoluted C 1s XPS spectrum of S-130-13, which reveals different chemical states in its graphene structure. The observed carbon chemical states include C=C sp^2^ (284.7 eV), C-C sp^3^ (285.4 eV), O-C-O (286.7 eV), O-C=O (288.5 eV), and π-π* satellite (291.5 eV) [35,36,37]. The π-π* satellite peak at 291.5 eV is indicative of aromatic or conjugated systems typically observed in graphene structures [36,37]. The predominance of the C=C sp^2^ peak in the deconvoluted C 1s spectrum suggests a successful conversion of PI to LIG, effectively suppressing the other peaks associated with C-C sp^3^, O-C-O, and O=C-O. The chemical structure of the S-130-13 electrode is also examined using Raman spectroscopy, as shown in Figure 4h. The Raman spectrum of S-130-13 reveals three prominent peaks, namely D, G, and 2D, located at approximately 1350, 1580, and 2700 cm^−1^, respectively. The D peak corresponds to defects or bent sp^2^ carbon, while the G peak represents the first-order scattering vibration of sp^2^ carbon atoms and the 2D peak originates from second-order zone boundary phonons [38]. The quality of graphene formation can be assessed through the intensity ratios of the peaks (Table S2, Supporting Information): the ratio of the 2D peak to the G peak (*I*_2*D*_/*I_G_*) indicates the degree of graphene formation, and the ratio of the D peak to the G peak (*I_D_*/*I_G_*) reflects structural disorder within the graphene [1,11]. Additionally, the XRD pattern of S-130-13 shows a peak at 25.8°, corresponding to the (002) plane in the LIG, indicating a high degree of graphitization, as illustrated in Figure 4i. Another peak at 42.6° reflects the (100) plane, highlighting the in-plane structure [1].

### 3.2. LIG Formed Through a Double-Lasing Process

**Figure 5:**
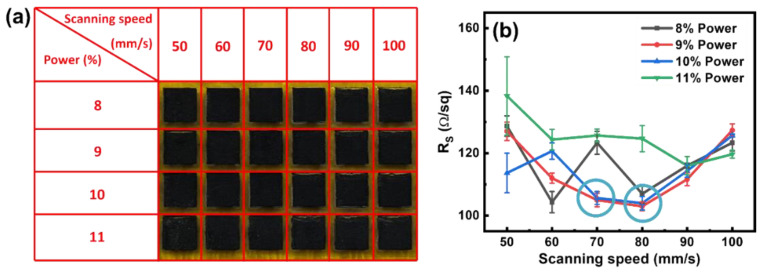
(**a**) Physical appearance of LIG formed through double-lasing conditions under a range of lasing conditions. (**b**) Sheet resistance (R_S_) of the LIG as a function of scanning speed and power used.

After optimizing the conditions for the formation of LIG through a single-lasing process, a second lasing was conducted on S-130-13. Specifically, following the initial lasing on a PI film to form S-130-13, the sample was subjected to another lasing process with various combinations of laser power and scanning speed. In the initial attempts, S-130-13 underwent a second lasing process under conditions similar to that used for the first lasing (i.e., 130 mm/s and 13% power). However, the formed LIG film completely peeled off, likely due to the high power level. To prevent this issue, we adjusted the second lasing settings to use lower power and scanning speed, ensuring the formed LIG layers remained intact. The power was modified from 8 to 11%, while the scanning speed was adjusted between 50 and 100 mm/s. Figure 5a illustrates that the formed LIG maintained on PI films, indicating their stable physical appearance after the second lasing irradiation. In addition, we investigated the changes in sheet resistance (R_S_) of the LIG after the second lasing, as shown in Figure 5b. The R_S_ values of the LIG samples irradiated at 11% power were noticeably higher than those at 8, 9, and 10%, which is attributable to the over-oxidation of the LIG film. For the lower power levels of 9 and 10%, R_S_ values decreased as the scanning speed increased to 70 or 80 mm/s. This is presumably because the thermal accumulation at these speeds was not significant enough to degrade the LIG while still being sufficient to convert the remaining carbon-based components on the PI film into graphene, resulting in a reduction in R_S_. We selected four LIG samples with the lowest R_S_ values for a further evaluation of their electrochemical performance, namely D-70-9, D-70-10, D-80-9, and D-80-10, which are marked by blue circles in Figure 5b. The R_S_ values for these samples were 105.0, 105.7, 103.0, and 104.0 Ω/sq, respectively.

**Figure 6:**
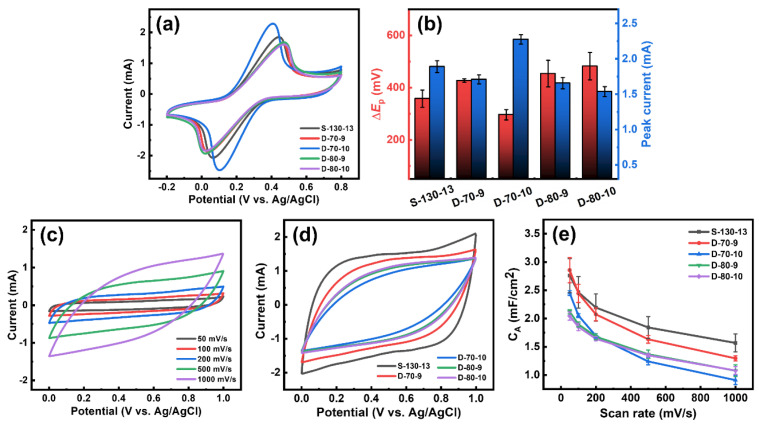
(**a**) CVs of 10 mM K_3_[Fe(CN)_6_] in 1 M KCl for D-70-9, D-70-10, D-80-9, and D-80-10 at a scan rate of 50 mV/s. For comparison, the CV obtained under the same condition for S-130-13 is also presented. (**b**) Peak-to-peak separation (Δ*E*_P_) and peak current values obtained from the CVs for each LIG. (**c**) CVs of D-70-10 in 1 M Na_2_SO_4_ at scan rates from 50 to 1000 mV/s. (**d**) CVs of D-70-9, D-70-10, D-80-9, and D-80-10 at a scan rate of 1000 mV/s. For comparison, the CV obtained under the same condition for S-130-13 is also presented. (**e**) Specific areal capacitance (C_A_) calculated from the CVs for each LIG as a function of scan rates.

Figure 6a shows CVs of four LIG samples, D-70-9, D-70-10, D-80-9, and D-80-10, which were formed using a double-lasing process. It also includes the CV of S-130-13 for comparison. The CVs revealed well-defined redox peaks of [Fe(CN)_6_]^3−^ on the LIG electrodes. To assess the electrochemical characteristics of the four LIG samples in relation to S-130-13, we evaluated their peak-to-peak separation (Δ*E*_P_) and peak currents, which reflect the electrochemical characteristics of the LIG samples. D-70-10 exhibited a significant decrease of ca. 60 mV in Δ*E*_P_ (296.4 mV) compared to S–130–13 (357.8 mV), while other double-lasing LIG samples showed higher values. Additionally, as shown in Figure 6b, the peak current value of D-70-10 was greater than those of S-130-13 and the other double-lasing LIG samples. These observations align with those of Δ*E*_P_ values, confirming the improved electrochemical performance of D-70-10. This demonstrates the potential of the double-lasing process for the facile fabrication of LIG electrodes with enhanced electrochemical characteristics, even without complicated chemical modifications of LIG. It is worth noting that low-quality LIG samples with high sheet resistance (R_S_) were produced with additional lasing iterations compared to double-lasing LIG, such as D-70-10 (Figure S4, Supporting Information). This observation indicates over-oxidation and the deterioration of LIG with additional lasing iterations after the second irradiation.

Figure 6c shows CVs of D-70-10 at various scan rates, ranging from 50 to 1000 mV/s. For additional details, the CVs of other double-lasing LIG samples are presented in Figure S1c–e (Supporting Information). The quasi-rectangular shapes of these voltammograms indicate the electrical double-layer capacitive behaviors of D-70-10 and the other double-lasing LIG samples, similar to what was observed with S-130-13. Figure 6d presents the CVs of all four double-lasing LIG samples, namely D-70-9, D-70-10, D-80-9, and D-80-10 alongside S-130-13, all recorded at a scan rate of 1000 mV/s. The quasi-rectangular symmetry of the voltammograms remained stable at this high scan rate, indicating the high stability of the LIG, which is essential for energy storage applications. Figure 6e demonstrates the correlation between C_A_ of the double-lasing LIG samples and scan rates. We observed an inverse trend, where the C_A_ values decreased as the scan rates increased. This can be attributed to the insufficient time for electrolyte ions to penetrate the porous structures of the LIG at high scan rates. The C_A_ value of D-70-9 is higher than those observed with other double-lasing LIG samples but is slightly lower than that of S-130-13. Specifically, at lower scan rates of 50 to 200 mV/s, the C_A_ values of D-70-9 remained similar to those of S-130-13. However, at higher scan rates of 500 and 1000 mV/s, the C_A_ values of D-70-9 gradually diminished. At a scan rate of 1000 mV/s, the C_A_ value of D-70-9 decreased to 45.1% of the value obtained at a lower rate of 50 mV/s, dropping from 2.86 to 1.29 mF/cm^2^. In contrast, S-130-13 maintained relatively high capacitance, retaining 56.9% of its value at 50 mV/s, decreasing from 2.76 to 1.57 mF/cm^2^. This demonstrates the adverse effect of second-lasing irradiation during a double-lasing process on the capacitive characteristics of LIG electrodes. Meanwhile, D-70-10 only retained 37.1% of its capacitance from 2.45 to 0.91 mF/cm^2^ after increasing the scan rate from 50 to 1000 mV/s, respectively. Even though it shows better electrochemical performance in terms of Δ*E*_P_ and peak current value, its capacitance can only be retained at 37.1%, which is lower than those of other double-lasing samples. Table S1 (Supporting Information) compares the C_A_ values of S-130-13 and D-70-9 with previously reported values, indicating that the electrochemical performance of the LIG electrodes is superior or at least comparable to those of chemically modified LIG found in the literature.

**Figure 7:**
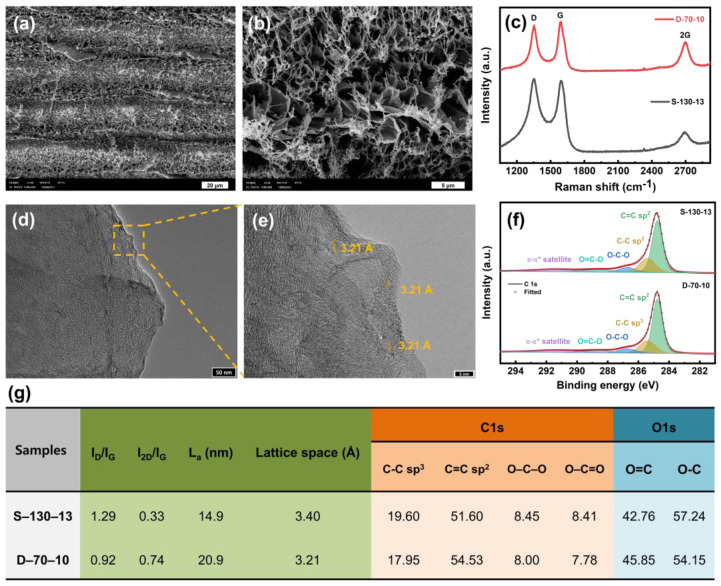
SEM images of D-70-10 at different magnifications of (**a**) 1000× and (**b**) 5000×. (**c**) Raman spectrum of D-70-10. For comparison, the Raman spectrum of S-130-13 is also presented. (**d**,**e**) TEM images of D-70-10 at different magnifications. The scale bar in panel (**d**) measures 50 nm, while the scale in panel (**e**) measures 5 nm. (**f**) Deconvoluted C 1s XPS spectra of S-130-13 and D-70-10. (**g**) Summary of differences in chemical structure between S-130-13 and D-70-10.

Figure 7a,b shows SEM images of D-70-10, which demonstrates that the double-lasing LIG featured three-dimensional interconnected graphene fibers and sheets, forming a porous structure like that of S-130-13. Additional SEM images of other double-lasing LIG samples are presented in Figure S2e–j (Supporting Information). The results indicate that there are no significant changes in morphology after the second lasing irradiation during the double-lasing process. Figure 7c shows Raman spectra of S-130-13 and D-70-10. The characteristic peaks of graphene, denoted as D, G, and 2D peaks, are observed at ~1350, ~1580, and ~2700 cm^−1^, respectively. The *I_D_*/*I_G_* ratio of 1.29 from S-130-13 indicates a highly defective nature caused by single-lasing irradiation [24]. The disordered structure and defects were reduced after the second lasing process during the double-lasing process, indicated by a decrease in the *I_D_*/*I_G_* ratio to 0.92 for D-70-10. Meanwhile, we observed an inverse trend in the *I*_2*D*_/*I_G_* ratio, which serves as an indicator of the number of graphene layers. A lower *I*_2*D*_/*I_G_* ratio indicates multilayer structures with reduced sp^2^ graphitization [1,17,18]. Thus, the increase in the *I*_2*D*_/*I_G_* ratio from 0.33 to 0.74 with the double-lasing process verifies that there are fewer graphene layers with a higher level of graphitization in the D-70-10 compared to S-130-13 formed with the single-lasing irradiation [39]. This can be attributed to the fact that the additional lasing process enhances the graphitization conversion of other carbon-containing components on the PI film. The enhanced graphitization consequently improves the electrochemical performance of LIG regarding the peak current and Δ*E*_P_ values. However, the reduction in graphene layers restricts the access of electrolyte species to the porous structure of LIG. This limitation could explain why the C_A_ value of D-70-10 is lower than that of S-130-13, as discussed above. Table S2 (Supporting Information) compares the *I_D_*/*I_G_* and *I*_2*D*_/*I_G_* values of D-70-10 and S-130-13 with previously reported values, demonstrating consistent and comparable results to previous research. Additionally, the crystallite size (*L_a_*), calculated using Equation (1), increased from 14.9 nm for S-130-13 to 20.9 nm for D-70-10, verifying a higher degree of graphitization caused by the double-lasing process [20]. Furthermore, Figure 7d,e shows TEM images of D-70-10, revealing a smaller lattice spacing of ~3.2 Å in contrast to S-130-13, which has a lattice spacing of ~3.4 Å. The slight decrease in lattice spacing observed after the second lasing process can be attributed to a reduction in oxygen-containing functional groups in S-130-13. This reduction allows the graphene sheets to pack more tightly, resulting in a smaller interlayer distance compared to the less reduced LIG samples [40,41]. XPS measurements were also performed to analyze the surface compositions and the oxidation states of S-130-13 and D-70-10 to verify the alterations following the double-lasing process. The XPS survey spectra of S-130-13 and D-70-10 show a decrease in oxygen content after the double-lasing process (Figure S3a, Supporting Information), which aligns with the TEM results indicating a reduction in the lattice spacing of D-70-10 to ~3.2 Å compared to S-130-13. In addition, as shown in Figure 7f, the deconvoluted C 1s XPS spectra of S-130-13 and D-70-10 reveal an increase in C=C sp^2^ and a decrease in C–C sp^3^ by ~3% and ~2%, respectively. This suggests that the double-lasing process facilitates the conversion of C–C sp^3^ to C=C sp^2^. A reduction in oxygen-containing functional groups was also observed in D-70-10, with O–C–O and O=C–O decreasing by a total of ~1% compared to S-130-13. Figure 7g summarizes differences in the chemical structure between S-130-13 and D-70-10.

**Figure 8:**
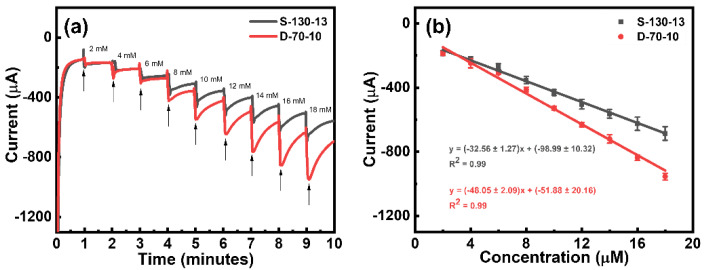
(**a**) Chronoamperometric responses of D-70-10 and S-130-13 upon the addition of different concentrations of H_2_O_2_ into N_2_-saturated 0.1 M phosphate buffer (pH 7.4) at −0.7 V. (**b**) Corresponding calibration curves of H_2_O_2_ for D-70-10 and S-130-13.

Thus far, we have investigated the relationship between laser irradiation and the electrochemical properties of LIG throughout the lasing processes and demonstrated the improved properties of double-lasing LIG compared to single-lasing LIG. Furthermore, we explored the possibility of using double-lasing LIG such as D-70-10 for developing electrochemical sensors. Specifically, we demonstrated the versatility of D-70-10 by utilizing their improved electrochemical properties for a sensitive amperometry analysis of H_2_O_2_ as a model analyte. Figure 8a shows the chronoamperometric responses of D-70-10 during the electrochemical reduction of H_2_O_2_ at an applied potential of −0.7 V. For comparison, the corresponding responses of S-130-13 are also included. Their electrochemical reduction currents increased linearly with successive additions of different concentrations of H_2_O_2_ into N_2_-saturated 0.1 M phosphate buffer (pH 7.4) at −0.7 V, which is consistent with the previous literature [20,42,43]. Figure 8b illustrates the linear relationship between the reduction currents and H_2_O_2_ concentrations, demonstrating the reliable and consistent responses of both D-70-10 and S-130-13 LIG electrodes. Both electrodes exhibited a high correlation coefficient of 0.99 when responding to varying concentrations of H_2_O_2_. Importantly, D-70-10 exhibited a significantly higher sensitivity of 48.05 μA/mM in contrast to S-130-13, which had a sensitivity of 32.56 μA/mM. This higher sensitivity of D-70-10 can be attributed to its improved electrochemical properties when compared to S-130-13.

## 4. Conclusions

Our work demonstrated the effect of the double-lasing irradiation process on the morphology and electrochemical features of the LIG electrodes. The fabrication of LIG electrodes was conducted by laser irradiation on a PI film substrate, and their electrochemical performance was investigated, focusing on sheet resistance (R_S_), specific areal capacitance (C_A_), peak-to-peak separation (Δ*E*_P_), and peak current. Although their morphology did not show significant changes between single-lasing and double-lasing irradiations, the graphitization level increased in double lasing compared to single lasing, as confirmed by the increase in the *I*_2*D*_/*I_G_* ratio from 0.33 to 0.74. This increase in the graphitization level led to a reduction in R_S_ and Δ*E*_P_, along with an increased peak current feature. We envision that these findings will facilitate the use of double-lasing processes in the fabrication of LIG with enhanced properties, eliminating the need for any chemical treatments.

## Data Availability

The original contributions presented in this study are included in the article/Supplementary Materials. Further inquiries can be directed to the corresponding author.

## Supplementary Materials

The following supporting information can be downloaded at: https://www.mdpi.com/article/10.3390/nano15050333/s1, Figure S1: CVs of (**a**) S-100-12, (**b**) S-120-12, (**c**) D-70-9, (**d**) D-80-9, and (**e**) D-80-10 in 1 M Na_2_SO_4_ at scan rates from 50 to 1000 mV/s; Figure S2: SEM images of (**a**,**b**) S-100-12, (**c**,**d**) S-120-12, (**e**,**f**) D-70-9, (**g**,**h**) D-80-9, and (**i**,**j**) D-80-10; Figure S3: (**a**) XPS survey spectra of S-130-13 and D-70-10. The inset summarizes atomic composition of C 1s and O 1s for S-130-13 and D-70-10. (**b**) Deconvoluted O 1s XPS spectra of S-130-13 and D-70-10. The inset summarizes detailed atomic composition of O 1s for S-130-13 and D-70-10; Figure S4: Sheet resistance (R_S_) of LIG as a function of the number of lasing processes. The inset is an optical image of LIG obtained after different numbers of lasing processes; Table S1: Summary of C_A_ values from the present study and the previously reported literature; Table S2: Summary of *I_D_*/*I_G_* and *I*_2*D*_/*I_G_* values from the present study and the previously reported literature. References [44,45,46,47,48,49] are cited in the supplementary materials.
