# Supplementary material for: Laser Irradiation and Property Correlation in Double-Lasing Processes on Laser-Induced Graphene Electrodes
Source: Nanomaterials (Basel). 2025 Feb 21;15(5):333. doi: 10.3390/nano15050333 (PMC11901533; doi:10.3390/nano15050333)
Supplement: Supplementary file 1 [file nanomaterials-15-00333-s001.zip › nanomaterials-3495573-supplementary.pdf]

Supporting Information

Laser Irradiation and Property Correlation in Double-Lasing Processes on Laser-Induced Graphene Electrodes

Tran Quoc Thang[1], and Joohoon Kim[1,2,*]

[1]Department of Chemistry, Research Institute for Basic Sciences, Kyung Hee University, Seoul 02447, Republic of Korea.

[2]KHU-KIST Department of Converging Science and Technology, Kyung Hee University, Seoul 02447, Republic of Korea.

*To whom correspondence should be addressed.

E-mail: jkim94@khu.ac.kr; Voice: +81-2-961-9384; Fax: +81-2-966-3701

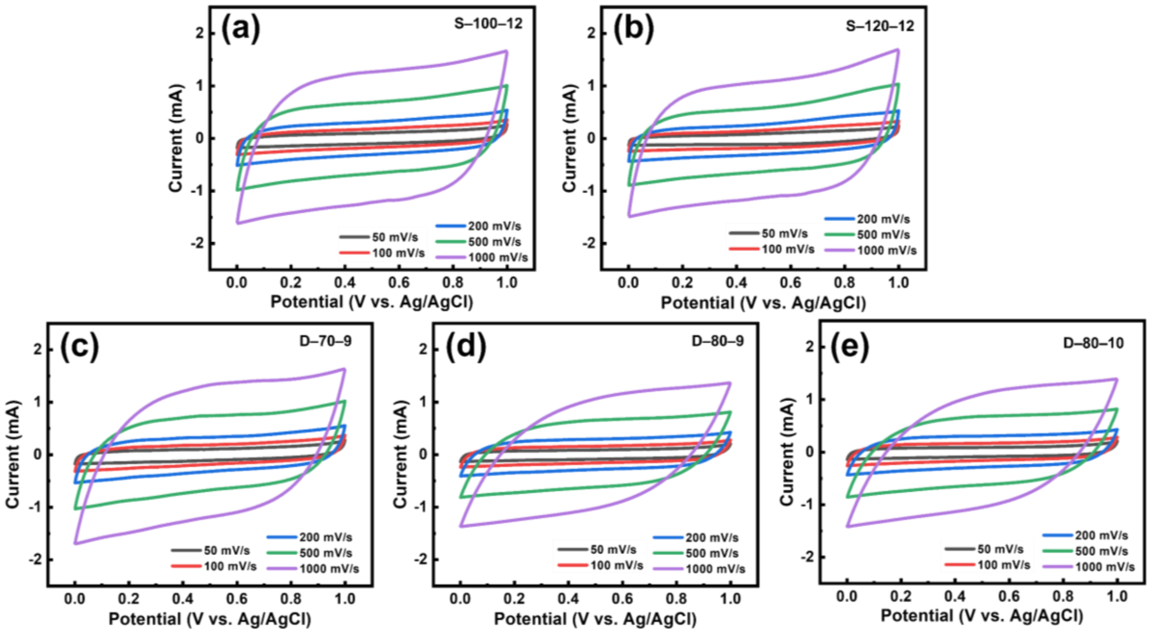

Figure S1. CVs of (a) S–100–12, (b) S–120–12, c) D–70–9, (d) D–80–9, and (e) D–80–10 in 1 M Na_2SO_4 at scan rates from 50 to 1000 mV/s.

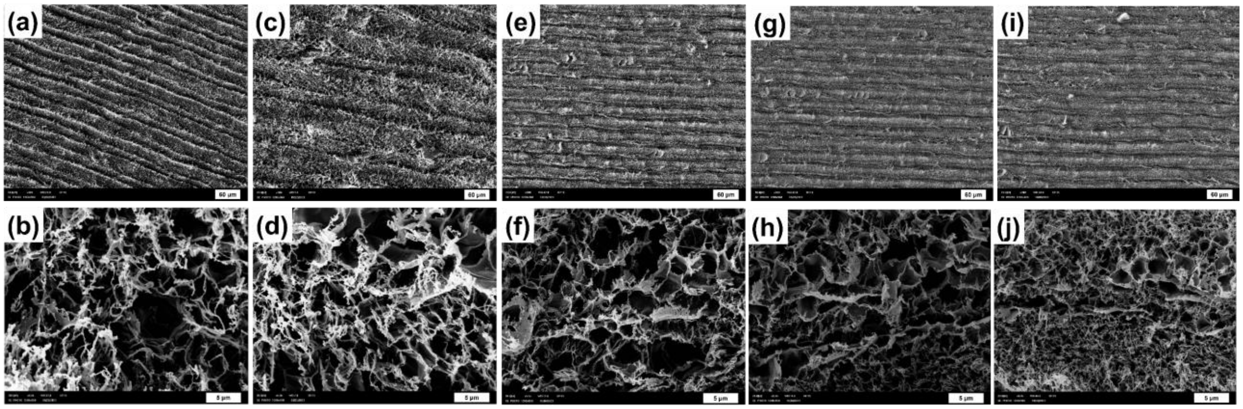

Figure S2. SEM images of (a, b) S–100–12, (c, d) S–120–12, (e, f) D–70–9, (g, h) D–80–9, and (i, j) D–80–10.

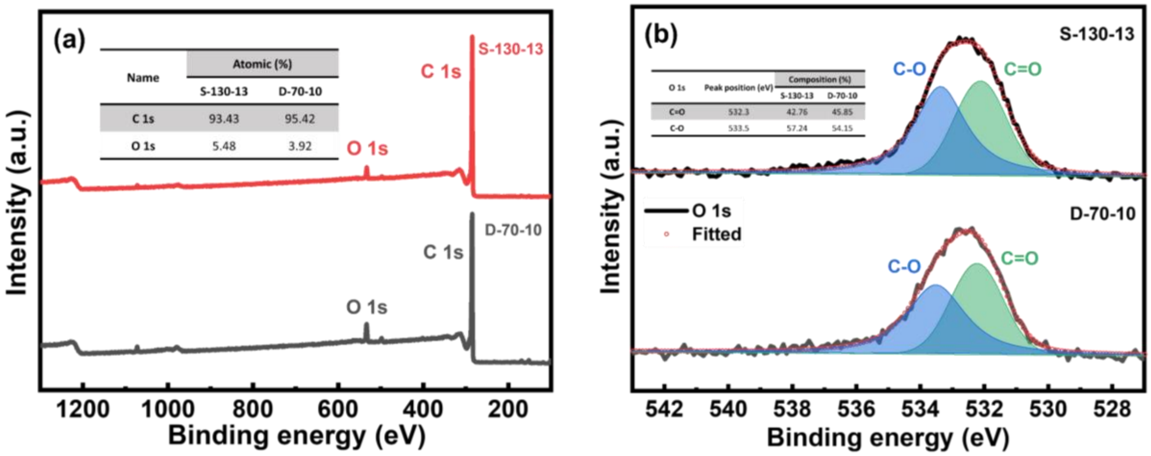

Figure S3. (a) XPS survey spectra of S–130–13 and D–70–10. The inset summarizes the atomic composition of C 1s and O 1s for S–130–13 and D–70–10. (b) Deconvoluted O 1s XPS spectra of S–130–13 and D–70–10. The inset summarizes the detailed atomic composition of O 1s for S–130–13 and D–70–10.

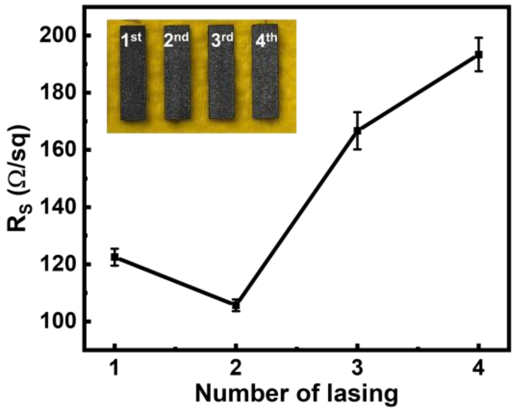

Figure S4. Sheet resistance (R_S) of LIG as a function of the number of lasing processes. The inset is an optical image of LIG obtained after different numbers of lasing processes.

Table S1. Summary of C_A values from the present study and the previously reported literature.

Sample	Substrate	Specific capacitance (C_A, mF/cm^2)	Electrolyte	Scan rate (mV/s)	Reference
LIG–MSCs	PI film	>4	1 M H_2SO_4	20	[1]
LIG/PDMS	PI film	0.65	PVP/NaCl	50	[2]
LSG–P24	Lignin	15.4	PVA/H_2SO_4	10	[3]
Laser–sculptured carbide	PI film	2	PVA/LiTFSI	100	[4]
MOF–199@ZIF–67	Quartz	2.5	1 M H_2SO_4	1000	[5]
LIG–MSCs	PI film	1.7	BMIMPF6–PVDF–HFP	5	[6]
LIG–MSC	PI foil	1.75	[EMIM][OTf]	5	[7]
LIG–HfO_2	PI film	2.8	PVA/H_2SO_4	40	[8]
LIG–MSCs	PI film	7.12	PVA/H_2SO_4	5	[9]
S–130–13	PI film	2.76 ± 0.30	1 M Na_2SO_4	50	This work
D–70–9	PI film	2.86 ± 0.22	1 M Na_2SO_4	50	This work

Table S2. Summary of I_D/I_G and I_{2D}/I_G values from the present study and the previously reported literature.

Substrate (Sample)	I_D/I_G	I_{2D}/I_G	Reference
PI film	0.44	0.88	[1]
PI/PDMS	0.72	N.A.	[10]
Paper	0.88	0.53	[11]
Paper	0.93	0.84	[12]
Chromatography paper	0.62	1.28	[13]
PI film	N.A.	0.36	[14]
PI film (S–130–13)	1.29	0.33	This work
PI film (D–70–10)	0.92	0.74	This work

References

1. Lin, J., et al., *Laser-induced porous graphene films from commercial polymers.* Nat. Commun., 2014. **5**, 5714.
2. Lamberti, A., et al., *A Highly Stretchable Supercapacitor Using Laser-Induced Graphene Electrodes onto Elastomeric Substrate.* Adv. Energy Mater., 2016. **6**, 1600050.
3. Zhang, W., et al., *Lignin Laser Lithography: A Direct-Write Method for Fabricating 3D Graphene Electrodes for Microsupercapacitors.* Adv. Energy Mater., 2018. **8**, 1801840.
4. Zang, X., et al., *Laser-sculptured ultrathin transition metal carbide layers for energy storage and energy harvesting applications.* Nat. Commun., 2019. **10**, 3112.
5. Zhang, W., et al., *Laser-Assisted Printing of Electrodes Using Metal–Organic Frameworks for Micro-Supercapacitors.* Adv. Funct. Mater., 2021. **31**, 2009057.
6. Shi, X., et al., *One-Step Scalable Fabrication of Graphene-Integrated Micro-Supercapacitors with Remarkable Flexibility and Exceptional Performance Uniformity.* Adv. Funct. Mater., 2019. **29**, 1902860.
7. Ray, A., J. Roth, and B. Saruhan, *Laser-Induced Interdigital Structured Graphene Electrodes Based Flexible Micro-Supercapacitor for Efficient Peak Energy Storage.* Molecules, 2022. **27**, 329.
8. Sain, S., et al., *Sputtered thin film deposited laser induced graphene based novel micro-supercapacitor device for energy storage application.* Sci. Rep., 2024. **14**, 16289.
9. Liu, T., et al., *High-performance micro supercapacitor assembled by laser-induced graphene electrode and hydrogel electrolyte with excellent interfacial wettability for high capacitance.* J. Power Sources, 2024. **602**, 234307.
10. Parmeggiani, M., et al., *PDMS/Polyimide Composite as an Elastomeric Substrate for Multifunctional Laser-Induced Graphene Electrodes.* ACS Appl. Mater. Interfaces, 2019. **11**, 33221-33230.
11. Bhattacharya, G., et al., *Disposable Paper-Based Biosensors: Optimizing the Electrochemical Properties of Laser-Induced Graphene.* ACS Appl. Mater. Interfaces, 2022. **14**, 31109-31120.
12. Park, H., et al., *Electronic Functionality Encoded Laser-Induced Graphene for Paper Electronics.* ACS Appl. Nano Mater., 2020. **3**, 6899-6904.
13. Pinheiro, T., et al., *Laser-Induced Graphene on Paper toward Efficient Fabrication of Flexible, Planar Electrodes for Electrochemical Sensing.* Adv. Mater. Interfaces, 2021. **8**, 2101502.
14. Johnson, Z.T., et al., *Electrochemical Sensing of Neonicotinoids Using Laser-Induced Graphene.* ACS Sens., 2021. **6**, 3063-3071.
